# General practitioners’ views on polypharmacy and its consequences for patient health care

**DOI:** 10.1186/1471-2296-14-119

**Published:** 2013-08-15

**Authors:** Juliane Köberlein, Mandy Gottschall, Kathrin Czarnecki, Alexander Thomas, Antje Bergmann, Karen Voigt

**Affiliations:** 1 Department of Health Care Management and Public Health, Schumpeter School of Business and Economics, University of Wuppertal, Wuppertal, Germany; 2 Department of General Practice / Medical Clinic III, Dresden Medical School, University of Technology, Fetscherstr. 74, Dresden 01307, Germany

**Keywords:** Polypharmacy, Multimorbidity, General practitioner, Health services research

## Abstract

**Background:**

Multimorbidity is defined as suffering from coexistent chronic conditions. Multimorbid patients demand highly complex patient-centered care which often includes polypharmacy, taking an average of six different drugs per day. Adverse drug reactions, adverse drug events and medication errors are all potential consequences of polypharmacy. Our study aims to detect the status quo of the health care situation in Saxony’s general practices for multimorbid patients receiving multiple medications. We will identify the most common clinical profiles as well as documented adverse drug events and reactions that occur during the treatment of patients receiving multiple medications. We will focus on exploring the motives of general practitioners for the prescription of selected drugs in individual cases where there is evidence of potential drug-drug-interactions and potentially inappropriate medications in elderly patients. Furthermore, the study will explore general practitioners’ opinions on delegation of skills to other health professions to support medical care and monitoring of patients receiving multiple medications.

**Methods/design:**

This is a retrospective cross sectional study using mixed methods. Socio-demographic data as well as diagnoses, medication regimens and clinically important events will be analyzed retrospectively using general practitioners documentation in patients’ records. Based on these data, short vignettes will be generated and discussed by general practitioners in qualitative telephone interviews.

**Discussion:**

To be able to improve outpatient health care management for patients receiving multiple medications, the current status quo of care, risk factors for deficient treatment and characteristics of concerned patients must be investigated. Furthermore, it is necessary to understand the physicians’ decision making process regarding treatment.

## Background

Patient age is correlated with the probability of having coexistent chronic conditions, known as multimorbidity, which requires highly complex patient-centered care [1]. Although there is no generally accepted definition of multimorbidity, it is obvious that the coexistence of several chronic diseases often affects patients’ quality of life. Furthermore, communities are confronted with socioeconomic and medical challenges concerning the whole health care sector [2]. Health care expenditures are correlated with the intensive care of patients within the last six months of life, over-medication and polypharmacy [3,4]. Regarding this, medication management could reduce health care expenditures [5-8].

In their literature review, Fortin et al. analyzed multimorbidity prevalences between 49 and 99% for individuals older than 65 years, living in the USA [9-11], Netherlands [12,13], Israel [14] and Canada (Québec) [15]. For these purposes, multimorbidity was defined as the existence of two or more chronic diseases [16]. For Germany there are only a few population-based studies concerning the prevalence of multimorbidity [17-19]. In 2011, van den Bussche et al. published a study based on health insurance data showing that 62% of 120,000 insured individuals over the age of 65 had more than three chronic diseases [20]. The Department of General Practice of the Dresden University of Technology conducted an epidemiological study (SESAM-4) which showed that 62% of 2,529 Saxon patients treated by a general practitioner (GP) suffered from multimorbidity (again defined as having two or more documented chronic conditions [21]). The CONTENT-Project (CONTinuous morbidity registration Epidemiologic NeTwork) of the University of Heidelberg offered data from more than 30,000 patients who were treated by GPs. The patients were predominantly from a western region of Germany (Baden-Württemberg) and confirmed a positive correlation between their age and the number of chronic diseases. Furthermore the investigation showed that the number of chronic diseases has a significant influence on the number of medications taken [22].

Overall, 20% of GPs’ patients over 65 receive 60% of all prescribed drugs [23]. Consequently, polypharmacy is one important aspect that should be considered in the management of multimorbid patients. In a national survey Kaufman et al. found that 57% of women over 65 living in the United States take more than five prescription medications [24]. A European study showed that 51% of participating patients take more than six prescribed medications per day [25]. Scientists from the German PRISCUS-Network observed that those patients take on average six different drugs per day. The number of drugs was significantly higher in patients over 65. The PRISCUS-Network consists of institutes, hospitals and groups working in health care, is promoted by the Federal Ministry of Education and Research, and focuses on investigating issues concerning the drug regimen on multimorbid elderly [26].

An analysis of the pharmacotherapy and medicine consulting service for physicians by the Association of Statutory Health Insurance Physicians in Saxony showed that patients take on average 4.7 drugs simultaneously [27]; individual patients were shown to take up to 21 drugs. In the framework of the federal government’s health monitoring it is pointed out that an individual should not take more than four drugs regularly [28]. Already five active ingredients that cause drug interactions are unclear and unpredictable [29,30], but regarding, for instance, cardiovascular diseases, the prescription of different active ingredients is clinically necessary and explains polypharmacy rates of 40% (including over-the-counter drugs) in patients over 75 [31].

In addition to potential adverse drug reactions (ADR) including interactions and adverse reactions, adverse drug events (ADE) and medication errors are further risks or rather potential consequences of polypharmacy [32-34]. ADE appear in approximately 13% of medicamentous and out-patient treated individuals and cause - besides individual health effects - a high economic burden [35], which is estimated at 800 million Euros per year for Germany [36]. Older people are particularly affected by ADE, as multimorbidity is associated with age [37]. Medication or dosage errors as well as non-compliance occur far more frequently and result from unclear dosage and medication instructions or complex disease management [38]. Moreover, self-medication means that physicians have a limited overview and control of adequate medication management. In Germany and in a large number of other European countries, the GP usually manages the whole treatment including medication management of multimorbid patients.

Based on polypharmacy and age-related pharmacokinetic and pharmacodynamic changes, the risk of ADR and ADE is increasing in elderly patients [39]. There are several drugs for elderly patients for which the risks of intake outweigh the benefits, known as potential inappropriate medications (PIM). Building on this, a few lists with recommendations or guidelines for managing appropriate medication in elderly has been developed, such as the Beers criteria [40] or the STOPP criteria [41].

As potential inappropriate medication and prescriptions occur frequently among the elderly in Germany, a PRISCUS-Network study was performed. Eighty three potentially inappropriate drugs for elderly typical in the German drug market were identified and summarized in the PRISCUS-list [23]. Due to the complex pharmacotherapy of geriatric patients, such instruments may support patient-centered care with the aim of avoiding inappropriate medication. Therefore listed drugs are increasingly integrated in guidelines. Although using the PRISCUS-list can improve the medical care of the elderly it has still some deficiencies. The list includes advice concerning comorbidities but it does not focus on multimorbidity or polypharmacy [23]. Further projects focusing on improving the treatment of multimorbid patients in Germany are predominantly done in the context of trans-sectoral cooperation [42-44]. Preliminary results show that through this cooperation, physicians are able to detect inadequate medication and potential ADE early within the treatment process, reduce adverse events and improve patients’ knowledge as well as behavior.

Against this background we will conduct a study that focuses on polypharmacy in multimorbid patients in GPs’ practices. We intend to determine the status quo of medication of multimorbid patients as well as different patterns of polypharmacy in general practices in Saxony. Furthermore, we want to explore motives for GPs’ medical prescription in case of prescription of potentially inappropriate medication. We aim to create scientific evidence that helps to develop a sustainable and region-specific care management. Compared to other German federal states, Saxony has the highest percentage of patients over 65 (24.7%) [45]. By 2020, the number of patients over 65 is expected to rise to 28.5%. Hence, in Germany one of the oldest population currently and in the near future will be in Saxony. By developing regional strategic solutions, Saxony could play a leading role for the other German federal states. Recent study results in the field of pharmacoepidemiology could identify regional differences in care, especially concerning medication [46-48]. In addition, the current German social law requires that patient-centered care should be improved by making it more regionally oriented and flexible [49].

Therefore, the study aims to detect the status quo of the health care situation in GPs’ practices for multimorbid patients receiving multiple medications in Saxony. In addition to the criteria of polypharmacy and multimorbidity we will also identify the most common clinical profiles and related ADE and ADR documented by the participating GPs (for example risk of falling, delirium and syncope). We aim to identify demands, obstacles and opportunities for improvement.

Summarized, the study will focus on following research questions:

(1) What is the current situation of health care in GPs’ practices concerning multimorbid patients receiving polypharmacy?

(2) Which problems appear during the treatment of patients receiving polypharmacy?

(3) What are the motives for GPs’ decision making regarding prescription of potentially inappropriate drugs?

(4) Which patient groups should be differentiated from GPs’ point of view, to address specific treatment processes correctly?

(5) May the delegation of medical work in GPs’ practices to other health professionals improve the care of multimorbid patients receiving multiple medications and what should be delegated to whom?

Based on this explorative study, hypotheses for regional concepts of patient-centered care under consideration of polypharmacy will be generated and should be tested in further studies.

## Methods

### Study design

**Figure 1:**
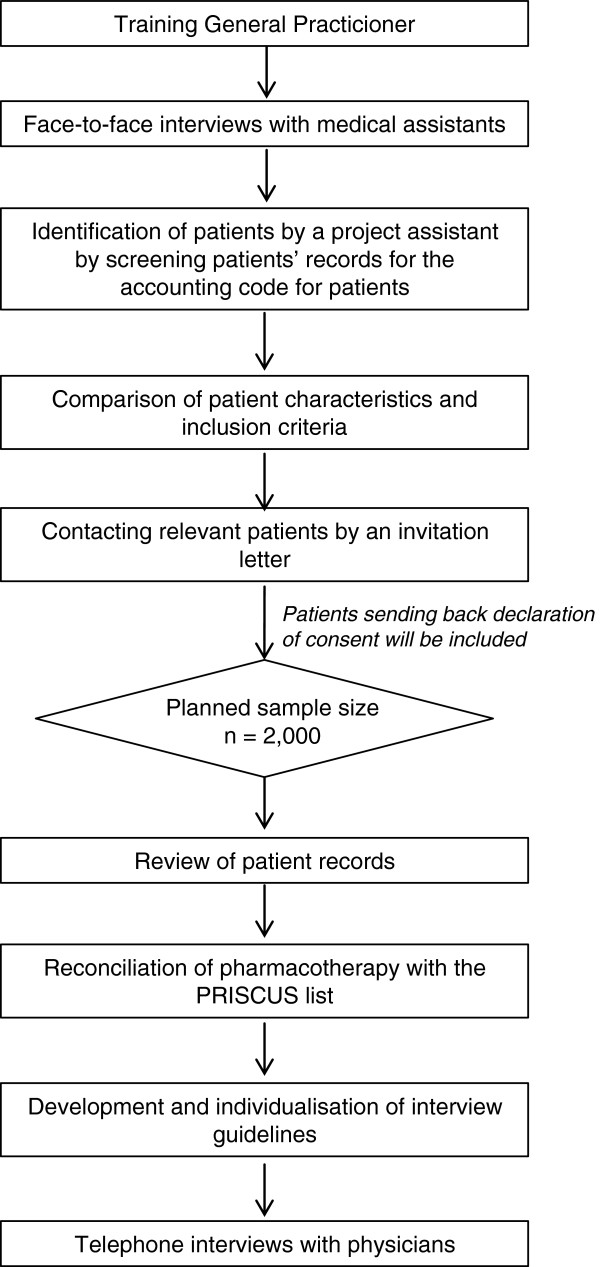
Study procedure.

This retrospective cross sectional study using mixed methods consists of three different research parts (Figure 1):

(1) Content analysis of patient records

(2) Interview with GPs including short vignettes that focus on prescription of PIM and polypharmacy

(3) Interview with medical assistants

Ethics approval for the study was obtained from the Ethics Commission of the Medical Clinic, Dresden University of Technology. Approval number: EK410122012.

### Setting

The study will be conducted in the setting of outpatient primary care in the urban region of Dresden. The academic teaching practices from the Department of General Practice of the Dresden University of Technology in Saxony will be invited for recruitment of GPs, medical assistants and patients.

### Recruitment of study participants

*GPs from academic teaching practices* (n ≈ 40) will be asked to participate. Considering the experience of previous research with academic teaching practices, we expect a response-rate of 25-30% [50]. The GPs will be contacted by an informative letter briefly explaining details of required data and the interview process. After written confirmation of participation in the study by interested GPs, a project assistant will visit the participating GPs personally to explain study design, provide study instruction and gain the GPs’ agreement to participate. In this context, a monetary incentive will be provided to positively influence participation and cooperation [51-53].

*Patients* will be recruited by a project assistant with support from the medical assistants and the GPs by screening patients’ records for the accounting code for patients with chronic diseases. This accounting code is defined by at least two consultations at GPs practice per quarter and at least one chronic disease for more than a year, which requires regular treatment. Based on this list of chronic patients, every patient will be checked for the following inclusion criteria: a) at least two parallel chronic diseases and b) two prescribed long-term medications at least six months before 2012. The invitation to participate in the study will contain a personal cover letter from the GP, information about the research study, the declaration of consent and a prepaid envelope for responding and will be dispatched by postal service to the included patients. Patients that send back their consent will be included for analysis. In case of incapacitated patients (such as demented persons), approval by custodians could be given.

Based on a prevalence of about 60% of multimorbid patients in general practice [20,21], we plan to include of 200 patients per practice. Thereby, the total sample results in approximately 2000 patients (10 practices, each with 200 patients).

### Research instruments

For the collection of data following instruments will be used:

(1) Semi-standardized content analysis (pseudonymous) of patient’s records using prepared analytical protocols

(2) Standardized questionnaire describing participating GPs and practices

(3) PRISCUS-list to compare the prescribed drugs for elderly patients

(4) Semi-standardized and guideline-based single telephone interviews with GP

(5) Semi-standardized and guideline-based single face-to-face interviews with medical assistants/receptionists

Semi-standardized instruments will be used to collect comparable data as well as to ensure openness toward unpredictable information.

Research instruments, strategies for recruitment of participants and data collection were tested within a pretest that was conducted by one of the co-operation partners with a GP practice in Wuppertal in February/March 2013.

(1) Content analysis (pseudonymous) of patient’s records

 Patient records will be retrospectively analyzed per patient and per quarter in 2012. The quarters will be assigned randomly to the patients. The analysis will be focused on a) determination of morbidity profiles, participation in chronic care programs and acute diseases, b) associated drug prescriptions, c) admission and referral to specialists and other health professionals, d) patients’ socio-demographicdata and number of consultations in GPs’ practice.

(2) Standardized questionnaire describing participating GPs and practices

 We will collect information on characteristics of the participating practices, for example type of practice, number of patients per quarter, age and duration of professional experience, delegation to medical assistants, proportion of elderly patients ≥ 65 years and experience with the PRISCUS-list.

(3) PRISCUS-list to compare the prescribed drugs for elderly patients

 We will compare the prescribed drugs with the PRISCUS-list to identify potentially inappropriate medication. Furthermore, we will explore examples for generating short vignettes that will be discussed in the interviews with GPs.

(4) Semi-standardized and guideline-based single telephone interviews with GP

 GPs will be interviewed to discuss the results of the patient`s records analysis and to understand their decision making in treatment of multimorbid patients. Thereby, the reasons for an exceptional prescription of a medication of the PRISCUS-list will be explored. Furthermore, it will be explored whether the GPs ask their patients regularly about self-medication, co-treating physicians or ADE. It is also important to determine if the patients receive any information material concerning ADE, ADR or drug administration.

(5) Semi-standardized and guideline-based single face-to-face interviews with medical assistants/receptionists

 Interviews will be performed with the medical assistants to gain information about patients’ frequently asked questions at the reception area of GPs’ practice. Additionally, we will focus on medication and related problems. Table 1 summarizes the study measures and their instruments.

**Table 1:** Summary of measures

**Measure**	**Patient record**	**Telephone interviews with physicians**	**Telephone interviews with medical assistants**	**Practice registration form**
**Structure of practice**				
Sociodemographic data of physicians				√
Number of years worked as a physician / medical assistant		√	√	√
Type of practice (e.g. single or community practice)				√
**Patient characteristics**				
Number of patients quartlerly				√
Sociodemographic data of patients	√			
Percentage of patients older than 65 years				√
Diagnosis of patient	√			
Patients participation in chronic care programs	√			
Patients number of doctor visits	√			
**Medication**				
Long-term and acute medication (e.g. dosage and active ingredients)	√			
Other therapies and interventions as well as their frequency and duration (e.g. physiotherapy, patient education and rehabilitation)	√			
Weighting of influencing factors during medication prescription		√		
Noncompliance of patients receiving polypharmacy		√		
Reports of adverse events, interactions or other consequences of polypharmacy		√	√	√
Describing typical patients, reporting problems with medication		√		
Taking account of recommendations in the PRISCUS-List				√
**Organization and processes in practice**				
Needed time for treating patients receiving polypharmacy		√	√	
Difficulties on patient admission			√	
Potential for changing practice organization to improve the treatment of patients receiving polypharmacy		√		√
Potential and difficulties concerning cooperation with pharmacies		√		
Use of individual case vignettes in practice		√		
Evidence of formal qualifications of medical assistants			√	√

### Data analysis

The collected data from the analysis of patients’ records will be documented pseudonymously via a case report form and transferred to a SPSS data matrix. After a plausibility check, the data will be analyzed descriptively (using 95% confidence intervals). Depending on dispersion and variance of the data we will use multivariate models to find predictors for several outcome parameters. The qualitative data gained by interviews will be categorically interpreted and summarized according to the qualitative content analysis of Mayring [54].

## Discussion

To support the goal of improving out-patient health care management for patients with multimorbidity, the status quo of care, risk factors for over-, under- and misuse of health services and the number as well as characteristics of concerned individuals must be investigated. This study will provide new evidence about the process of GPs’ decision making regarding the treatment of multimorbid patients.

Information gained by interviewing medical assistants will describe the process and content of a preliminary talk at the reception area of GPs’ practice. In case of patient-reported medication-related problems the medical assistants will give information about the kind of the problems and their handling with these issues. Regarding the limited resources in health care, it is moreover necessary to reflect existing delegation concepts and to examine their acceptance by GPs, medical assistants and patients. In future, it is necessary to analyze the effectiveness and costs of these concepts.

Nevertheless, there will be some limitations of our study that should be considered. The sample of teaching practices might be not representative for all GPs in Saxony [55]. There could be differences in non-teaching practices regarding prescription and managing treatment because of more evidence based knowledge based on regular teaching trainings. Concerning representativeness of the patient sample of research active GPs compared to non-active GPs, there are hints that there are no significant differences regarding demography, morbidity and mortality of the patients [56,57]. Despite the likely representativeness of our basic population of patients, the data could be biased by patients’ voluntary participation of the study (response bias). Furthermore, quality and quantity of patients’ records could differ between GPs. For example there will be known and unknown patients’ diseases, which will not be coded by the GPs as long-term diagnosis. Based on this, some patients will not apply to the inclusion criteria. Consequently, the prevalence of chronic conditions and multimorbidity would be underestimated. Summarized, interpretation of the results should be done with respect to the context and the setting. The qualitative parts of our study will help to detect and to understand similarities and differences in GPs’ acts.

## Abbreviations

ADE: Adverse drug events; ADR: Adverse drug reaction; GP(s): General practitioner(s); PIM(s): Potentially appropriate medication(s); PRISCUS: SPRerequISites for a new health Care model for elderly people with mUltiple morbidities; SESAM: Sächsische Epidemiologische Studie in der Allgemeinmedizin; CONTENT: CONTinuous morbidity registration Epidemiologic NeTwork.

## Competing interests

The authors declare that they have no competing interests.

## Authors’ contributions

JK, CZ, MG and KV collectively drafted the study protocol and sought funding and ethical approval. AB, MG and KV are responsible for the management of the study. AB and AT revised the manuscript of the paper. All authors read and approved the final manuscript.

## Pre-publication history

The pre-publication history for this paper can be accessed here:

http://www.biomedcentral.com/1471-2296/14/119/prepub
